# Supplementary material for: Development of methodology to support molecular endotype discovery from synovial fluid of individuals with knee osteoarthritis: The STEpUP OA consortium
Source: PLoS One. 2024 Nov 18;19(11):e0309677. doi: 10.1371/journal.pone.0309677 (PMC11573211; doi:10.1371/journal.pone.0309677)
Supplement: S1 Table — (DOCX) [file pone.0309677.s010.docx]

| Cohort Name and ethical approval number | City, Country | QC Sample Grouping^1^ | Disease Type/Severity/Surgical procedure | Total Samples Contributed^2^  N (n) | Samples submitted for QC^3^N (n) | Sex^4^, female n (%) | Age^4^, median (IQR) | Ordinal KL grade^4,5^, median (IQR) |
| --- | --- | --- | --- | --- | --- | --- | --- | --- |
| *KANON*  (LU 535-01, ISRCTN84752559) | Lund, Sweden | Acute knee Injury | Anterior cruciate ligament tear | 72 (44) | 72 (44) | 12 (16.7) | 24 (7) | 0 (0) |
| *KICK*  (10/H0805/39) | Oxford, UK | Acute knee Injury | Acute knee injury | 140 (140) | 140 (140) | 26 (18.6) | 25 (9.5) | 1 (2) |
| *OxKIC*  (14/WA/1108) | Oxford, UK | Acute knee Injury | Anterior cruciate ligament and/or meniscal injury | 33 (33) | 33 (33) | 8 (24.2) | 32 (13) | 0 (0) |
| *COASt*  (10/H0604/91) | Oxford, UK | OA | Knee arthroplasty | 20 (20) | 20 (20) | 13 (65) | 73 (13) | - |
| *MJC*  (2017-0183) | Maastricht, Netherlands | OA | Knee arthroplasty | 237 (219) | OA: 218 (200) | 115 (52.8) | 69 (14) | 3 (1) |
| *Distraction study*  (160/D; NL51539.041.15) | Utrecht, Netherlands | OA | Joint distraction | 38 (20) | 38 (20) | 13 (34.2) | 55 (8) | 3 (1) |
| *EARLY OA*  (REB 16-5969 AE) | Toronto, Canada | OA | Non-advanced disease | 43 (43) | 43 (43) | 19 (44.2) | 58 (13) | 2 (0) |
| *LEAP OA*  (16-5759 BE) | Toronto, Canada | OA | Knee arthroplasty | 339 (339) | 338 (338) | 189 (55.9) | 66 (12) | - |
| *LUMEN*  (LU 49-98) | Lund, Sweden | OA | Varying severity | 32 (32) | 32 (32) | 9 (28.1) | 62 (16) | - |
| *LUMEX*  (LU 361-00) | Lund, Sweden | OA | Varying severity | 67 (67) | 67 (67) | 34 (50.8) | 64 (10) | 3 (1) |
| *DBOARD*  (14/EM/0013) | Nottingham, UK | OA | Varying severity | 46 (46) | 46 (46) | 26 (56.5) | 68.5 (9) | 2 (3) |
| *MenTOR*  (15/SC/0551) | Oxford, UK | OA | Varying severity | 75 (75) | 75 (75) | 21 (28) | 47 (11) | 2 (1) |
| *NBS GOAL*  (EC2/06) | Nottingham, UK | OA | Varying severity | 112 (112) | OA: 74 (74) | 33 (44.6) | 67 (12) | 3 (1) |
|  |  | Non-OA controls | No radiographic disease |  | Non-OA controls: 31 (31) | 8 (25.8) | 65 (17) | 0 (0) |
| *OMB*  (09/H0606/11)  *MMOA*  (07/H0706/81) | Oxford, UK | OA / non-OA controls / injury | Arthroplasty | 199 (199) | OA: 194 (194) | 105 (54.1) | 69 (12) | - |
|  |  |  |  |  | Non-OA control(s): 1 (1) | 1 (100) | 46 (-) | - |
|  |  |  |  |  | Injury: 1 (1) | 0 | 29 (-) | - |
| *Molecular Pathways*  (20/EM/0065) | Nottingham, UK | OA | Varying severity | 45 (45) | 45 (45) | 27 (60) | 67 (10) | 2 (1) |
| *WebEx*  (18/EM/0154) | Nottingham, UK | OA | Varying severity | 20 (20) | 20 (20) | 13 (65) | 66 (13.5) | 2 (1) |
| *WOREO*  (REB 109255) | London, Canada | OA | Varying severity | 245 (207) | 236 (204) | 135 (57.2) | 65 (15) | 3 (2) |
| *Other:*  *NBS GOAL*  (EC2/06)  *Understanding pathogenesis of OA* (16/LO/1351)  Molecular Mechanisms  (07/H0706/81) | Nottingham, UK  Oxford, UK  Oxford, UK | OA / non-OA controls / inflammatory controls | - | 17 (15) | OA: 5 (5) | 2 (40) | 81 (3) | - |
|  |  |  |  |  | Non-OA controls: 5 (5) | 2 (40) | 64.5 (21.5) | - |
|  |  |  |  |  | Inflammatory controls: 5 (5) | 3 (60) | 47 (9) | - |

**S1 Table.** ***Characteristics of Cohorts and Participant Samples.***

^1^ QC sample grouping: a broad description of the participant’s membership, attributed by cohort-level information at baseline, of one of the following groups:

Knee OA, acute knee joint injury, non-OA (healthy) controls, inflammatory arthritis controls.

^2^N shown is the total number of SF samples contributed to STEpUP OA analysis by each cohort (total = 1780 samples) before sample exclusions prior to inclusion into the quality control (QC) analysis.

^3^In parentheses, n shown is the number of samples passing QC for each cohort (total = 1746 samples). 34 of 1780 (1.9%) samples were excluded from the QC analysis: 10 samples were excluded at processing stage, 18 samples were of insufficient volume to be processed by SomaLogic and 6 were contralateral knee samples from same participant to be used in later analyses.

^4^Demographic and basic clinical data for those samples passing QC analysis (1746 samples) at the time of SF sample collection are summarized.

^5^For the LUMEN cohort, whilst radiographic scores were available, these were not ordinal KL grades so not reported here.

Abbreviations: OA (osteoarthritis), KL (Kellgren and Lawrence), UK (United Kingdom), QC (Quality Control), Nonsurgical versus Surgical Treatment Study (KANON), Knee Injury Cohort at the Kennedy (KICK), Oxford Knee Injury Cohort (OxKIC), Clinical Outcomes in Arthroplasty Study (COASt), Lund Meniscus cohort (LUMEN), Osteoarthritis Biomarker Follow up study (D-BOARD consortium), MJC, Maastricht Joint Collection, Meniscal Tear and Osteoarthritis Risk (MenTOR), Nottingham Biomarker Study Genetics of Osteoarthritis and Lifestyle (NBS GOAL), Oxford Musculoskeletal Biobank (‘OA pathogenesis’ project) (OMB), Molecular Mechanisms in OA (MMOA), Western Ontario Registry for Early Osteoarthritis (WOREO) Knee Study.
