# Supplementary material for: Development of methodology to support molecular endotype discovery from synovial fluid of individuals with knee osteoarthritis: The STEpUP OA consortium
Source: PLoS One. 2024 Nov 18;19(11):e0309677. doi: 10.1371/journal.pone.0309677 (PMC11573211; doi:10.1371/journal.pone.0309677)
Supplement: S2 Table — (DOCX) [file pone.0309677.s011.docx]

| **Applied to** | **Field** | **Description** | **Coding** |
| --- | --- | --- | --- |
| **Quality Control  and Downstream Analysis** | **sf_iknee_sample_id_number** | The STEpUP OA Sample Identification Number (SIN) | string |
|  | **stepup_id** | The STEpUP OA Participant Identification Number(PIN) | string |
| **Quality Control** | **age_sampling** | Patient age at the time sample was taken (to the nearest year) | integer (NA=missing) |
|  | **sl_plate_id** | Identification (ID) of plate the sample was run on | string |
|  | **sl_plate_run_date** | Date that the sample was run | string (“dd-mm-yyyy”) |
|  | **sl_plate_position** | Position of the sample on the 96-well plate | string (“XN”, where X is row letter and N is the column number) |
|  | **sl_scanner_id** | ID of the SomaScan scanner that the sample was read on | string |
|  | **sl_tranche_number** | Shipment tranche in which sample was run | {1 = tranche 1, 2 = tranche 2} |
|  | **sl_bimodal_signal** | The technical bimodal signal, strongly correlated with processing batch, used to batch-correct the data. | {bimodal1, bimodal2 - arbitrary labels for the two groups. NA = missing} |
|  | **sf_iknee_proc_batch** | Batch number for index knee sample | Integer (NA = missing) |
|  | **sf_iknee_proc_order** | Processing order number for knee samples | Integer (NA = missing) |
|  | **sf_iknee_proc_treat_date** | Date sample was hyaluronidase-treated by Oxford | Text (dd-mm-yyyy) |
|  | **sf_iknee_qc_group** | Patient grouping (OA, acute knee injury or control) at baseline | {0 = OA, 1 = Joint injury, 2 = healthy control, 3 = inflammatory control, NA = missing} |
|  | **cohort_name** | Cohort ID (an arbitrarily chosen integer assigned to each cohort) | integer |
|  | **sex** | Patient sex at baseline (as defined by individual cohort collectors) | {m = male, f = female, NA = missing} |
|  | **sample_age** | Time between date of sample collection and date of STEpUP OA sample processing for the index knee (years) | float (years) (NA = missing) |
|  | **sf_iknee_volume** | Total SF volume collected (ml) | float (ml) |
|  | **sf_iknee_prev_freeze_thaw** | Whether the sample had been freeze-thawed prior to STEpUP OA sample processing | {0 = No, 1 = Yes, NA = Unknown} |
|  | **sf_iknee_freezethaw_cycles** | Number of freeze-thaw cycles (if known) | integer (NA=missing) |
|  | **sf_iknee_freezethaw_spec** | Whether the sample  has been freeze-thawed less than, or greater to or equal to five times | {0 = <5, 1 = ≥5, NA = missing} |
|  | **sf_iknee_bloodstaining** | Grading of SF bloodstaining prior to centrifugation (if known). Scale of 1-4, with larger numbers corresponding to greater degrees of blood staining (by visual inspection) | {1 = None, 2 = Mild, 3 = Moderate, 4 = Severe, NA = Not known} |
|  | **sf_spun_vs_unspun** | Indicator for whether the sample was centrifuged prior to being received at Oxford | 0 = unspun, 1 = spun, 2 = not known |
| **Downstream Analysis (Discovery Analysis & Replication Analysis)** | **Cohort name** | Cohort ID (an arbitrarily chosen integer assigned to each cohort) | integer |
|  | **Disease allocation** | Patient grouping (OA, acute knee injury or control) at baseline. Note that this estimate of disease was based primarily on the inclusion criteria of the individual cohorts, not at individual level | {0 = OA, 1 = acute knee injury, 2 = healthy control, 3 = inflammatory control, NA = missing} |
|  | **Age** | Patient age at the time sample was taken (to the nearest year) | integer (NA = missing) |
|  | **Sex** | Patient sex at baseline (as defined by individual cohort collectors) | {m = male, f = female, NA = missing} |
|  | **BMI** | Patient body mass index at the time the sample was taken (calculated from provided height and weight or directly provided by cohort collector, in that order of preference) | float (kg/m^2) |
|  | **Ordinal KL grade (worst affected compartment)** | Kellgren-Lawrence (KL) grade of radiographic severity at time of sampling | {0 = grade 0 (none), 1 = grade 1 (doubtful), 2 = grade 2 (minimal), 3 = grade 3 (moderate), 4 = grade 4 (severe), NA = Missing OR Not Known} |
|  | **Binary indicator for the presence/absence of radiographic knee OA** | Flag indicating whether the sample was taken from a patient with radiographic OA in the index knee, defined as a KL grade greater or equal to two at time of sampling | {0 = No (i.e. KL < 2), 1 = Yes (i.e. KL ≥2, NA = Missing OR Not Known} |
|  | **Binary indicator for the presence of advanced stage radiographic knee OA (KL scores 3-4)** | Flag indicating whether the sample was taken from a patient with advanced radiographic OA in the index knee, defined as a KL grade greater or equal to three at time of sampling | {0 = No (i.e. KL < 3), 1 = Yes (i.e. KL ≥3), NA = Missing OR Not Known} |
|  | **Smoking history** | Flag indicating whether the patient was a current or past smoker at the time of the baseline sample | {0 = No (i.e. never smoked), 1 = Yes (i.e. current smoker or past smoker), NA = missing or not available} |
|  | **baseline** | Flag indicating whether this sample is a baseline sample (each individual has one baseline sample) | {0 = No, 1 = Yes} |
|  | **Harmonised Knee Pain Score** | Binary flag indicating whether experienced knee pain is outside of the Patient Acceptable Symptom State (PASS) at the time of sampling (calculated from the KOOS pain subscale, the WOMAC pain subscale or knee VAS (knee-specific NRS/VAS or painDETECT VAS, in order of preference). | {0 = No (acceptable pain), 1 = Yes (unacceptable pain), NA = missing or Not Available} |
|  | **Harmonised Patient Reported Outcome Measure (PROM)** | The specific patient reported outcome measure used to derive a harmonised knee pain score | {1 = KOOS, 2 = WOMAC, 3 = Knee specific VAS/NRS,  4 = PainDETECT VAS, NA=missing} |
|  | **KOOS pain score** | KOOS pain subscore (calculated from full KOOS questionnaire results, or from combined subscore provided by cohort collectors, in that order of preference). Scale of 0-100, where 0 is the worst possible pain recordable. | Float |
|  | **WOMAC pain score** | WOMAC pain subscore (calculated from full WOMAC questionnaire results, or from combined subscore provided by cohort collectors, or derived from full KOOS questionnaire results, in that order of preference). Scale of 0-100, where 100 is the worst possible pain recordable. | Integer |
|  | **Knee-specific numeric rating score (NRS)** | Patient reported knee pain on a Numeric Rating Scale (0-10), where 10 is the worst pain imaginable | Float |
|  | **PainDETECT numeric rating score (NRS)** | Patient reported average pain score (over the last 4 weeks) from the painDETECT questionnaire. Scale of 0-10, where 10 is the worst pain imaginable | Integer |

**S2 Table.**  ***Core Clinical Phenotype Data used for Quality Control and Downstream Analyses.***

These data include sample information used to carry out quality assessment, as well as clinical phenotype data required for the downstream Discovery and Replication analyses. ‘Plate’ refers to that used to assay the sample at SomaLogic. ‘Batch’ refers to the membership of a group of sessional processing by the Oxford Lab. ‘Tranche’ relates to overall larger groupings of sample processing (four in total) carried out by the Oxford Lab, where processed samples from each group were shipped together to SomaLogic. Note that details of various pre-defined knee pain measures and harmonised scores are given here, but their analysis is not reported in this manuscript (variables were predefined for the discovery and replication analysis). This is also true for some of the measures of radiographic severity (KL grade is included here). Abbreviations: WOMAC, Western Ontario and McMaster Universities Osteoarthritis Index; KOOS, The Knee injury and Osteoarthritis Outcome Score; NRS, Numeric rating score; VAS; visual analogue scale; PROM, patient reported outcome measure; SF, synovial fluid; BMI, Body Mass Index.
