# Supplementary material for: Development of methodology to support molecular endotype discovery from synovial fluid of individuals with knee osteoarthritis: The STEpUP OA consortium
Source: PLoS One. 2024 Nov 18;19(11):e0309677. doi: 10.1371/journal.pone.0309677 (PMC11573211; doi:10.1371/journal.pone.0309677)
Supplement: S3 Table — (DOCX) [file pone.0309677.s012.docx]

| **Target** | **Target Full Name** | **Type of Assay** |
| --- | --- | --- |
| **TIMP-1** | Tissue Inhibitor of Metalloproteinase-1 | MSD Ultra-Sensitive |
| **MMP-3** | Matrix Metalloproteinase-3 (Stromelysin-1) | MSD Ultra-Sensitive |
| **IL-6** | Interleukin-6 | MSD V-PLEX |
| **IL-8** | Interleukin-8 | MSD V-PLEX |
| **MCP-1** | Monocyte Chemoattractant Protein-1 (C-C motif chemokine 2) | MSD V-PLEX |
| **FGF2** | Fibroblast growth factor 2 | MSD V-PLEX |
| **Activin A** | Activin A | R&D Quantikine ELISA |
| **TGFβ1** | Transforming growth factor Beta-1 | R&D Quantikine ELISA (active and latent combined) |
| **TSG-6** | Tumor necrosis factor-inducible gene 6 | MSD inhouse assay (U plex) |

**S3 Table. *Summary of Proteins Measured by Immunoassay used to assess accuracy of SomaScan Data.***

Nine protein targets included on the SomaScan platform (V4.1) had been previously measured using immunoassay of a subset of the synovial fluid samples (albeit not hyaluronidase treated), by either conventional sandwich ELISA (R&D) or by electrochemiluminescent assay (MSD) ("Type of Assay") following manufacturer’s instruction, and these data were used to evaluate the degree of agreement with the SomaScan data. Manufacturers were: MSD, Mesoscale Discovery, Rockville and R&D, R&D Systems, Minneapolis, both US.
