# Supplementary material for: Development of methodology to support molecular endotype discovery from synovial fluid of individuals with knee osteoarthritis: The STEpUP OA consortium
Source: PLoS One. 2024 Nov 18;19(11):e0309677. doi: 10.1371/journal.pone.0309677 (PMC11573211; doi:10.1371/journal.pone.0309677)
Supplement: S4 Table — (DOCX) [file pone.0309677.s013.docx]

| **Filter label** | **Filter Description** | **Description** | **Applies To** | **Excluded**  **Proteins/Samples**  **within**  **Non-IPS adjusted data** | **Excluded Proteins/Samples within**  **IPS adjusted data** |
| --- | --- | --- | --- | --- | --- |
| **NONHUMAN** | Non-human proteins | Non-human or control proteins | Proteins | 307 | 307 |
| **OA_REPO** | Reproducibility in OA pool | R^2^ < 0.5  (non-technical variation less than 50%) | Proteins | 485 | 485 |
| **INJ_REPO** | Reproducibility in acute knee injury pool | R^2^ < 0.5  (non-technical variation less than 50%) | Proteins | 252 | 252 |
| **FREEZETHAW_CONFOUND** | Associated with number of freeze-thaw cycles | ANOVA p < 0.05/7289  (conditional on cohort) | Proteins | 56 | 212 |
| **SAMPLEAGE_CONFOUND^1^** | Associated with sample age | ANOVA p < 0.05/7289  (conditional on cohort) | Proteins | 77 | 229 |
| **BIMODAL_CONFOUND** | Associated with bimodal signal | ANOVA p < 0.05/7289 | Proteins | 96 | 72 |
| **SOMASCAN_FAIL** | SomaLogic inhouse QC | Hybridization Scale Factor > 2.5 | Samples | 2 | 2 |
| **LOD_SAMPLE^2^** | Limit of detection | 25% of proteins below/above  limit of detection | Samples | 12 | 12 |
| **TOTPROT_OUTLIER** | Total protein outliers | >5 SDs from mean | Samples | 9 | 9 |
| **PCA_OUTLIER** | PCA outliers | >5 SD from combined centre on top PCs | Samples | 15 | 15 |
| **Total (number after filtering /number before filtering)** | | | Proteins | 6558/7596  (86.33%) | 6290/7596  (82.81%) |
|  |  |  | Samples | 1720/1746  (98.51%) | 1720/1746  (98.51%) |

**S4 Table.** ***Summary of Sample and Protein Filters.***

Details of the filters applied to the batch corrected, non-IPS adjusted data or IPS adjusted data, including thresholds used and number of samples or proteins (SOMAmers) removed by each filter. The final row gives the total number of proteins and samples remaining in the two datasets after filtering, to be used in downstream analyses. Note that proteins and/or samples removed were not mutually exclusive across filters. Abbreviations: IPS, intracellular protein score; PCA, Principal Component Analysis; SD, standard deviation. P values were Bonferroni corrected.

^1^Sample age was defined as the time from SF collection to time of SF processing at Oxford. ^2^Lower limit of detection (lLoD) was defined as the median concentration of the buffers plus 4.9 times the median absolute deviation of the buffers, and upper limit of detection (uLoD) was defined as 80,000 RFU, both as recommended by SomaScan platform.
