# Supplementary material for: Development of methodology to support molecular endotype discovery from synovial fluid of individuals with knee osteoarthritis: The STEpUP OA consortium
Source: PLoS One. 2024 Nov 18;19(11):e0309677. doi: 10.1371/journal.pone.0309677 (PMC11573211; doi:10.1371/journal.pone.0309677)
Supplement: S5 Table — (DOCX) [file pone.0309677.s014.docx]

| **IPS Adjustment** | **Predictor** | **Regression Coefficient** | **P-value** |
| --- | --- | --- | --- |
| **Non-IPS Adjusted Data** | ***Average protein abundance (log mean abundance)*** | ***-0.056*** | ***<2.23e-308*** |
|  | ***Non-secreted Nuclear protein (Y/N)^1^*** | ***0.033*** | ***1.64e-09*** |
|  | ***Non-secreted protein (Y/N) ^1^*** | ***0.051*** | ***3.98e-10*** |
|  | Monocyte protein (Y/N)^2^ | 0.033 | 0.077 |
|  | Neutrophil protein (Y/N)^2^ | 0.015 | 0.48 |
|  | Macrophage protein (Y/N)^2^ | -0.00044 | 0.98 |
| **IPS Adjusted Data** | ***Average protein abundance (log mean abundance)*** | ***-0.0080*** | ***5.8e-16*** |
|  | Non-secreted Nuclear protein (Y/N)^1^ | 0.012 | 0.25 |
|  | Non-secreted protein (Y/N) ^1^ | -0.019 | 0.23 |
|  | Monocyte protein (Y/N)^2^ | 0.038 | 0.30 |
|  | Neutrophil protein (Y/N)^2^ | -0.059 | 0.17 |
|  | Macrophage protein (Y/N)^2^ | 0.021 | 0.53 |

**S5 Table. *Predictors of the strength of correlation between protein abundance and PC1.***

Multiple linear regression results (coefficient and p-value) for the effect of a variety of protein-level factors on the correlation between each protein signal intensity and PC1. Protein abundance is calculated as the standardized RFU for each protein adjusted by the protein's dilution factor used in the SomaScan assay (the "dilution bin"). The dependent variable was the Pearson correlation between PC1 and protein signal intensity on a log scale. The independent variables were the average protein abundance on a log scale in SF, and flag indicating whether a protein was classified as a non-secreted protein (i.e. a protein not predicted to be secreted by the protein atlas) or a marker protein for monocytes, macrophages or neutrophils (taken from <https://panglaodb.se/index.html>), three common infiltrating immune cell types in the synovial joint. Results of log abundances and PC1 from the non-IPS adjusted and the IPS adjusted data are shown separately. Rows in bold italic show significant (p< 0.05) predictors, though all findings were also significant after Bonferroni correction for multiple testing. Abbreviations: IPS, intracellular protein score; SF, synovial fluid.
