# Supplementary material for: Development of methodology to support molecular endotype discovery from synovial fluid of individuals with knee osteoarthritis: The STEpUP OA consortium
Source: PLoS One. 2024 Nov 18;19(11):e0309677. doi: 10.1371/journal.pone.0309677 (PMC11573211; doi:10.1371/journal.pone.0309677)
Supplement: S6 Table — (DOCX) [file pone.0309677.s015.docx]

| **Protein Target** | **Activin A** | **FGF2** | **IL6** | **IL8** | **MCP1** | **MMP3** | **TGFb1** | **TIMP1** | **TSG6** |
| --- | --- | --- | --- | --- | --- | --- | --- | --- | --- |
| **Protein Full Name** | Activin A | Fibroblast growth factor 2 | Interleukin-6 | Interleukin-8 | Monocyte Chemoattractant Protein-1  (C-C motif chemokine 2) | Matrix Metalloproteinase- 3 (Stromelysin-1) | Transforming growth factor Beta-1 | Tissue Inhibitor of Metalloproteinase-1 | Tumor necrosis factor-inducible gene 6 |
| **OA** | 0.160  (0.324) | 0.250  (0.119) | 0.085  (0.604) | 0.381  (0.015) | 0.160  (0.323) | 0.112  (0.491) | 0.008  (0.964) | 0.204  (0.207) | 0.261  (0.104) |
| **Injury** | 0.481  (3.73e-2) | 0.683  (1.26e-3) | 0.585  (8.49e-3) | 0.639  (3.23e-3) | 0.598  (6.81e-3) | -0.073  (0.765) | 0.806  (3.06e-5) | 0.377  (0.112) | NA |

**S6 Table. *Correlation between Intracellular Protein Score and protein abundance measured by Immunoassay.***

Nine proteins measured on SomaScan were also measured on conventional immunoassay (see Supplementary S3 table for details of assays used). This table shows the correlation (correlation coefficients, with p-values in parentheses, both derived from Pearson correlation testing) between intracellular protein score and concentrations of key proteins measured on immunoassay, stratified by osteoarthritis (OA) and acute knee injury (Injury) samples. [Activin A, FGF2, IL6, IL8, MCP1, TGFb1 concentrations showed significant correlation with intracellular protein score. The estimated correlation coefficients were higher in injury samples compared with OA samples for 7 out of 8 proteins with data in both groups].
