# Supplementary material for: Development of methodology to support molecular endotype discovery from synovial fluid of individuals with knee osteoarthritis: The STEpUP OA consortium
Source: PLoS One. 2024 Nov 18;19(11):e0309677. doi: 10.1371/journal.pone.0309677 (PMC11573211; doi:10.1371/journal.pone.0309677)
Supplement: S7 Table — (DOCX) [file pone.0309677.s016.docx]

| **Proteomic Data** | **Confounders** | **Plate ID** | **Disease Group** | **Plate Position** | **Plate Run Date** | **Sample Processing Tranche** | **Sample Previous Freeze Thaw**  **(Y/N)** | **Sample Freeze Thaw Cycles** | **Sample Processing Batch** | **Date of Hyaluronidase Treatment** | **Sample Age** | **Sample Volume** | **Visual Blood Staining** |
| --- | --- | --- | --- | --- | --- | --- | --- | --- | --- | --- | --- | --- | --- |
|  | **PC (variation explained)** |  |  |  |  |  |  |  |  |  |  |  |  |
| **Standardised +**  **Bimodal Signal Adjustment +  IPS adjustment + Filtering** | PC1 (~17.0%) | 1.00E+00 | **8.98E-16** | **6.81E-05** | 9.48E-01 | **7.74E-04** | 1.96E-01 | 1.18E-01 | **1.81E-03** | **1.30E-04** | **2.81E-04** | **9.76E-04** | **5.97E-13** |
|  | PC2 (~6.9%) | 1.00E+00 | **1.01E-19** | 1.02E-02 | 9.44E-01 | 1.73E-02 | 2.32E-02 | 1.20E-01 | **1.61E-09** | **1.17E-04** | 3.55E-03 | **3.26E-04** | 7.44E-01 |
|  | PC3 (~4.8%) | 1.00E+00 | **2.58E-23** | **5.20E-04** | 9.74E-01 | **4.41E-11** | **2.74E-05** | 3.27E-01 | **4.32E-39** | **3.19E-15** | **1.28E-03** | **9.95E-23** | 5.39E-03 |
|  | PC4 (~4.0%) | 1.00E+00 | 3.24E-02 | **1.02E-05** | 9.62E-01 | 2.70E-01 | 8.02E-01 | 5.80E-01 | **4.33E-16** | **3.17E-07** | **2.67E-03** | 5.66E-01 | 1.30E-01 |
|  | PC5 (~3.4%) | 1.00E+00 | **8.18E-28** | 9.56E-02 | 9.72E-01 | 2.51E-01 | 6.68E-01 | 2.90E-01 | **3.06E-15** | **2.07E-04** | 9.63E-01 | **2.77E-06** | **1.70E-34** |
|  | PC6 (~2.9%) | 1.00E+00 | **1.20E-05** | **3.35E-03** | 9.82E-01 | **3.33E-06** | **9.88E-09** | **2.58E-08** | **1.25E-32** | **3.85E-21** | 9.08E-03 | **7.15E-05** | 5.10E-03 |
|  | PC7 (~2.4%) | 1.00E+00 | **3.72E-07** | **4.21E-37** | 9.32E-01 | 4.70E-01 | 4.78E-02 | 5.14E-01 | **1.69E-10** | **3.90E-05** | 1.85E-01 | 3.95E-01 | 1.66E-01 |
|  | PC8 (~2.0%) | 1.00E+00 | **9.35E-48** | 3.27E-02 | 9.80E-01 | **1.01E-05** | **7.35E-04** | 7.12E-01 | **5.93E-33** | **2.05E-14** | **4.69E-04** | 7.52E-01 | 8.69E-02 |
|  | PC9 (~1.7%) | 1.00E+00 | **1.24E-08** | **4.57E-56** | 9.51E-01 | 8.67E-01 | 6.46E-01 | 9.07E-01 | **1.08E-07** | 5.96E-02 | 9.97E-01 | 9.41E-01 | 5.72E-01 |
|  | PC10 (~1.5%)­ | 1.00E+00 | **8.92E-43** | **4.24E-11** | 8.24E-01 | **8.49E-07** | 1.28E-01 | 6.08E-01 | **5.88E-14** | **1.25E-08** | **2.82E-03** | 1.56E-02 | **1.76E-03** |
| **Standardised +**  **Bimodal Signal Adjustment +  Filtering  (without IPS adjustment)** | PC1 (~49.0%) | 1.00E+00 | **2.58E-12** | 6.90E-02 | 8.07E-01 | **2.70E-02** | 9.54E-01 | 1.63E-01 | **5.82E-05** | **1.36E-05** | 1.03E-01 | 2.50E-01 | **2.14E-25** |
|  | PC2 (~5.0%) | 1.00E+00 | **6.02E-40** | **3.10E-02** | 9.97E-01 | **2.08E-04** | 3.18E-02 | 9.32E-02 | **7.22E-03** | **1.38E-02** | 7.05E-02 | **3.91E-09** | **5.09E-11** |
|  | PC3 (~3.6%) | 1.00E+00 | **1.51E-10** | 1.62E-01 | 8.91E-01 | **2.38E-03** | **1.71E-03** | 8.52E-01 | **8.93E-23** | **7.55E-09** | **2.13E-10** | **3.49E-11** | 2.63E-01 |
|  | PC4 (~2.6%) | 1.00E+00 | **8.41E-11** | **1.83E-08** | 9.94E-01 | **1.79E-06** | 8.79E-02 | 8.63E-02 | **5.07E-18** | **2.60E-09** | 8.80E-01 | **1.12E-05** | **1.59E-02** |
|  | PC5 (~2.1%) | 1.00E+00 | **1.62E-05** | 7.73E-01 | 9.57E-01 | **1.26E-03** | **1.38E-04** | **5.43E-05** | **2.54E-95** | **7.29E-20** | **2.80E-04** | 1.67E-01 | **2.89E-05** |
|  | PC6 (~1.6%) | 1.00E+00 | **1.30E-36** | **1.43E-02** | 9.34E-01 | 4.78E-02 | 9.04E-01 | 6.36E-01 | **4.11E-14** | **1.40E-08** | 3.78E-02 | **5.08E-08** | **2.05E-31** |
|  | PC7 (~1.5%) | 1.00E+00 | 6.89E-02 | **3.01E-04** | 9.34E-01 | 2.77E-01 | **4.37E-05** | **3.35E-03** | **2.58E-24** | **1.65E-09** | 2.13E-01 | **1.80E-02** | 1.07E-01 |
|  | PC8 (~1.3%) | 9.98E-01 | **1.79E-06** | **1.05E-46** | 8.78E-01 | 4.30E-01 | **4.64E-04** | 7.68E-02 | **1.62E-06** | **6.18E-05** | 2.22E-01 | 7.70E-02 | 6.66E-02 |
|  | PC9 (~1.1%) | 1.00E+00 | **5.03E-76** | **2.72E-02** | 9.09E-01 | **1.15E-04** | **3.52E-03** | 2.49E-01 | **1.71E-17** | **2.03E-10** | **3.87E-06** | 5.25E-02 | 1.11E-01 |
|  | PC10 (~0.9%) | 1.00E+00 | **4.21E-23** | **6.46E-34** | 9.54E-01 | 1.07E-01 | 4.42E-01 | 7.40E-01 | **3.54E-06** | **1.08E-02** | 2.86E-01 | 1.76E-01 | 9.79E-01 |
| **Standardised +**  **Bimodal Signal Adjustment +  IPS adjustment  (without Filtering)** | PC1 (~16%) | 1.00E+00 | **9.75E-13** | **2.96E-04** | 9.98E-01 | **3.10E-04** | 3.32E-01 | 1.27E-01 | **1.04E-03** | **8.20E-05** | **2.20E-04** | **6.21E-03** | **1.25E-10** |
|  | PC2 (~8.1%) | 1.00E+00 | **6.61E-07** | **1.79E-04** | 9.96E-01 | **2.28E-05** | 3.62E-01 | 9.32E-02 | **5.19E-12** | **3.75E-06** | 1.51E-01 | 5.40E-01 | 9.80E-01 |
|  | PC3 (~5.1%) | 1.00E+00 | **9.23E-25** | 1.73E-01 | 1.00E+00 | **3.22E-10** | **5.85E-08** | 1.48E-01 | **5.56E-67** | **2.00E-21** | **1.48E-07** | **4.60E-20** | **8.73E-08** |
|  | PC4 (~4.5%) | 1.00E+00 | **1.46E-19** | 9.43E-01 | 9.92E-01 | 5.10E-01 | 5.38E-01 | **1.27E-04** | **1.90E-28** | **3.22E-06** | 5.02E-02 | **2.94E-04** | **1.17E-03** |
|  | PC5 (~3.6%) | 1.00E+00 | **1.32E-08** | **4.06E-07** | 9.86E-01 | 9.56E-01 | 4.60E-02 | 4.13E-02 | **2.00E-08** | **8.61E-04** | 1.46E-01 | 6.44E-02 | **1.05E-13** |
|  | PC6 (~3.1%) | 1.00E+00 | **1.09E-06** | **5.32E-23** | 9.86E-01 | **3.50E-03** | **5.83E-06** | **2.28E-04** | **2.25E-09** | **4.53E-12** | 1.04E-01 | **5.67E-08** | **1.04E-11** |
|  | PC7 (~2.6%) | 1.00E+00 | **6.96E-06** | **4.54E-11** | 9.85E-01 | 1.35E-02 | 5.67E-01 | 6.90E-01 | **7.14E-21** | **5.27E-07** | 2.34E-01 | 1.16E-01 | **2.69E-06** |
|  | PC8 (~2.2%) | 1.00E+00 | **9.18E-56** | **5.48E-33** | 9.88E-01 | 1.46E-02 | **2.19E-03** | 2.41E-02 | **1.57E-14** | **1.62E-09** | **6.87E-04** | 2.93E-01 | **1.34E-04** |
|  | PC9 (~1.9%) | 1.00E+00 | 1.17E-01 | **4.79E-31** | 9.87E-01 | 4.82E-01 | 4.95E-02 | 3.22E-01 | **4.02E-11** | **7.81E-03** | 1.46E-01 | 9.02E-01 | 7.49E-01 |
|  | PC10 (~1.6%) | 1.00E+00 | **1.50E-29** | **1.63E-19** | 9.52E-01 | **3.43E-03** | 3.81E-01 | 8.57E-01 | **1.07E-05** | **7.05E-05** | 3.56E-02 | 1.22E-01 | **1.96E-06** |
| **Standardised +**  **Bimodal Signal Adjustment  (without IPS adjustment without filtering)** | PC1 (51.0~%) | 1.00E+00 | **1.22E-10** | 9.69E-02 | 1.00E+00 | 2.23E-01 | 8.66E-01 | 2.17E-01 | **4.93E-04** | **2.72E-04** | 1.61E-01 | 9.28E-01 | **1.80E-27** |
|  | PC2 (~4.6%) | 1.00E+00 | **6.07E-19** | 9.79E-02 | 9.99E-01 | **2.04E-05** | 5.21E-02 | 2.72E-02 | **1.40E-05** | **5.34E-03** | 5.98E-01 | **3.62E-05** | **3.71E-07** |
|  | PC3 (~3.6%) | 1.00E+00 | **1.93E-05** | **4.46E-06** | 9.40E-01 | **1.41E-02** | 1.80E-01 | 4.83E-01 | **3.55E-12** | **2.52E-07** | **1.79E-07** | 5.91E-01 | **3.36E-05** |
|  | PC4 (~3.2%) | 1.00E+00 | **2.88E-29** | **3.26E-05** | 1.00E+00 | **2.33E-07** | **1.42E-03** | 4.29E-01 | **3.19E-36** | **5.32E-12** | **5.56E-05** | **1.03E-17** | 3.80E-02 |
|  | PC5 (~2.6%) | 1.00E+00 | **2.42E-05** | 5.36E-01 | 9.67E-01 | **2.94E-04** | **2.80E-04** | **6.72E-05** | **1.84E-88** | **2.13E-21** | **1.13E-02** | 7.59E-01 | **2.84E-03** |
|  | PC6 (~1.5%) | 1.00E+00 | **1.43E-05** | **1.79E-02** | 9.82E-01 | **9.78E-03** | **1.72E-02** | 7.43E-01 | **1.68E-38** | **4.32E-14** | **1.75E-03** | 3.95E-01 | 1.18E-01 |
|  | PC7 (~1.4%) | 1.00E+00 | **2.73E-46** | 5.02E-02 | 9.73E-01 | 4.58E-02 | 5.44E-01 | 9.88E-01 | **1.88E-11** | **1.77E-05** | 4.01E-01 | **1.41E-08** | **1.57E-32** |
|  | PC8 (~1.4%) | 9.99E-01 | **6.34E-15** | **2.27E-147** | 9.34E-01 | 6.10E-01 | **7.36E-03** | 7.17E-01 | 9.03E-02 | **2.49E-02** | 5.81E-01 | 5.90E-01 | 9.55E-02 |
|  | PC9 (~1.2%) | 1.00E+00 | **2.18E-31** | **5.87E-08** | 9.53E-01 | 2.78E-02 | **5.06E-07** | **8.49E-06** | **1.36E-14** | **2.14E-09** | **3.73E-05** | **1.14E-04** | 1.05E-01 |
|  | PC10 (~0.9%) | 1.00E+00 | **2.16E-05** | **5.83E-08** | 9.96E-01 | **1.63E-02** | 4.68E-01 | 3.59E-01 | **6.26E-09** | **2.54E-03** | 2.25E-02 | **9.32E-03** | 6.95E-01 |
| **Standardised  Data** | PC1 (48%) | **8.71E-07** | **2.00E-09** | 3.41E-01 | **1.27E-02** | 4.84E-01 | 8.63E-01 | 2.15E-01 | **1.38E-03** | **7.53E-04** | 5.94E-02 | 9.22E-01 | **4.31E-29** |
|  | PC2 (7.2%) | 2.15E-01 | **4.92E-10** | 5.73E-01 | 8.88E-02 | **1.15E-07** | 2.61E-01 | 1.65E-01 | **<2.23e-308** | **1.95E-56** | **4.50E-12** | 9.37E-01 | 7.48E-02 |
|  | PC3 (4.5%) | **3.70E-03** | **1.41E-04** | 4.91E-02 | 2.90E-01 | 1.99E-01 | 4.35E-01 | 3.96E-02 | **3.80E-04** | 9.21E-02 | 1.31E-01 | 7.76E-02 | 9.18E-02 |
|  | PC4 (3.6%) | **4.35E-02** | **2.44E-13** | 1.27E-01 | 6.09E-02 | **1.88E-02** | 8.88E-02 | **1.78E-02** | **1.85E-12** | **3.72E-04** | 6.55E-01 | **6.54E-06** | **8.65E-16** |
|  | PC5 (2.9%) | **3.59E-09** | **3.13E-31** | 2.07E-01 | 8.85E-01 | **2.19E-03** | **1.62E-02** | **1.86E-02** | **1.82E-16** | **4.38E-06** | **2.71E-03** | **1.48E-14** | 3.15E-01 |
|  | PC6 (1.6%) | **7.62E-09** | **3.12E-08** | 2.35E-01 | 5.40E-02 | **2.63E-10** | **3.44E-04** | 5.45E-02 | **1.41E-34** | **1.54E-13** | 7.17E-01 | **6.94E-03** | **1.42E-05** |
|  | PC7 (1.3%) | **5.99E-09** | **1.48E-22** | **5.17E-04** | **2.69E-03** | **1.54E-02** | 8.47E-01 | 5.10E-01 | **2.15E-10** | **1.11E-04** | **1.51E-02** | **3.64E-06** | **2.63E-25** |
|  | PC8 (1.3%) | **1.28E-49** | **1.62E-04** | **5.71E-49** | **1.97E-06** | 4.71E-01 | **8.60E-06** | **6.14E-03** | **3.77E-06** | **6.37E-06** | 3.62E-02 | **8.15E-03** | **6.18E-03** |
|  | PC9 (1.1%) | **9.52E-05** | **2.91E-76** | **4.13E-12** | **1.96E-03** | **6.28E-03** | **1.47E-02** | **2.13E-05** | **1.86E-07** | **1.56E-06** | **1.08E-04** | 1.06E-01 | **3.08E-07** |
|  | PC10 (0.9%) | **7.52E-08** | **2.29E-09** | **6.19E-11** | **5.66E-05** | **1.41E-02** | 1.21E-01 | 6.18E-02 | **6.38E-10** | **1.43E-03** | 2.59E-01 | 3.54E-02 | 5.45E-01 |

**S7 Table. *Associations between technical confounders and top 10 PCs.***The impact of technical confounders on each of the top 10 PCs of the two final sets of log abundance data (batch corrected, non-IPS adjusted and filtered, and batch corrected, IPS adjusted and filtered), measured using p-values from linear regression. In each case, the principal component was the dependent variable, and the technical variable was the independent variable, with some technical variables treated as linear predictors (number of freeze thaw cycles, sample age, sample volume, visual blood straining grade) and the rest treated as categorical variables. For technical variables that differed systematically by cohort (tranche number, previous freeze-thaw, number of freeze-thaw cycles, processing batch, treatment date, sample age, sample volume and blood staining), we included cohort as a covariate. Further details on the technical variables used assessed and their definitions are shown in S2 table. Entries with Benjamini-Hochberg adjusted p< 0.05 are bolded. Sample refers to samples of synovial fluid. Abbreviations: PC, principal component; IPS, intracellular protein score.
