# Supplementary material for: Development of methodology to support molecular endotype discovery from synovial fluid of individuals with knee osteoarthritis: The STEpUP OA consortium
Source: PLoS One. 2024 Nov 18;19(11):e0309677. doi: 10.1371/journal.pone.0309677 (PMC11573211; doi:10.1371/journal.pone.0309677)
Supplement: S1 File — (DOCX) [file pone.0309677.s017.docx]

**Supplementary Methods**

**Consortium structure and governance**

Founding members of the STEpUP OA Consortium signed the Consortium Agreement in October 2019 and associated Data Sharing Agreement (see Consortium membership). An accession document allowed new joiners. The consortium was led by the University of Oxford (principal lead, Vincent), with synovial fluid (SF) samples and linked proteomic and clinical data being received, processed and held there. Proteomic analysis was by SomaLogic, Boulder, Colorado, US. Distinct working groups oversaw key activities according to pre-defined Terms of Reference (available on request) (S1 Fig). Individuals analysing the data within the Data Analysis Group additionally signed a memorandum of understanding. A webpage included a lay summary and video (https://www.kennedy.ox.ac.uk/oacentre/stepup-oa/stepup-oa).

**Ethics and cohorts**

University of Oxford Medical Sciences Central University Research Ethics Committee (CUREC) granted ethical approval for the processing, storage and use of samples and linked data for this project on 1^st^ November 2019 (R67029/RE001). Each site ensured compliance with local ethical and data protection policies and appropriate written informed consent from each participant via existing approvals which included collaborative use. All signed a material transfer agreement prior to movement of samples/data.

To ensure confidentiality and to generate a single dataset, secondary pseudo-anonymisation was performed by the Oxford site for all received samples and their associated data records, with secure records of linkage with the originating cohort’s participant identification numbers. Specifically, a STEpUP participant ID number (PIN) and related unique sample identification number (SIN) were generated for each participant and associated sample(s), which did not identify cohort.

**Laboratory methods**

***Local cohort SF collection and processing*:** SF was collected directly from the joint, typically by needle arthrocentesis. Typical time from collection of sample to temperature-controlled centrifugation (at 20 °C to prevent increase in viscosity, at least 1800G or above, ideally 3000G) was two hours. For centrifuged samples, only the supernatants were removed and stored. Samples were stored as 500 ul sub-aliquots in standard 2 ml cryovials in monitored ultra-low temperature freezers (-80°C), until transfer on dry ice by temperature-controlled shipping to Oxford for central processing. Duration of sample storage by the local cohort was recorded and used within the analysis. Samples with blood contamination were not excluded, but the blood content was graded and quantified (see S2 Table legend).

***Central laboratory SF processing, Oxford*:** (see also main methods) A single stock of the same lot number of bovine testicular HAse (type I-S, 618.4 U/mg; Cat. No. H3506 400-1000units/mg, Sigma Aldrich) was purchased for the project. Sufficient volume for the whole project of hyaluronidase stock solution was made at the start of the project, reconstituted with sterile 1x PBS (ThermoFisher, Cat No. 10010023) and stored in cryovials as 1.9 ml aliquots (sufficient to treat 10 SF samples) at -80°C until use.

Tranches were processed upon receipt in Oxford, and their processing order recorded. Sample numbers processed in Tranches 1-4 were 435, 610, 691, and 10 samples respectively. Once thawed, samples and the appropriate number of hyaluronidase aliquots were kept in pre-cooled blocks until use (Corning CoolRack, Cat no. CLS432052, Sigma-Aldrich). Positive displacement ‘Piston’ pipettes (MR-250, Anachem, Cat No. 17008579) with disposable capillary piston tips (Pos-D Tips 250 μL Prstrl 180/3 C-250, Anachem, Cat No. 17008608) were used to ensure accurate measurement of synovial fluid. Following enzyme treatment (see Methods), subaliquots of the hyaluronidase treated (HT) supernatants were made, so that 175 ul could be shipped to SomaLogic (these aliquots had suffix, HT1 added to sample SIN), with back up aliquots held at Oxford (HT2, HT3). Treated aliquots for each Tranche were held in temperature monitored freezers (-80°C) until transfer to SomaLogic.

***Preparation of pooled SF samples.*** Pooled samples of SF were prepared for QC purposes, as outlined in Methods. Sufficient pooled SF samples were prepared and processed at the start of the project to be able to send to SomaLogic sufficient HAse treated pooled sample to include one pooled OA SF sample and one pooled post-injury SF sample on every plate. Each pool (4.5 ml total volume) included a mixture of 750 ul SF from each of six participants with OA or knee injury respectively. In sample selection, resentative ages and sex were considered as well as volumes available to contribute to the pool. For the unspun pooled sample, a lower total volume of 936 ul, included a mixture of 156 ul from each of six participants who had matched spun samples. These pools were all subaliquotted into 190 ul aliquots and enzyme treated on the same day by the same method at the start of the Project, using the project’s HAse stock batch. In addition, 4 OA pooled aliquots were stored untreated. One aliquot of each of these was subsequently thawed and enzyme treated at the same time as each new Tranche (2-4) of SF samples, to consider any change of activity of long-term storage of prepared HAse over time. Furthermore, one OA pooled sample was freeze-thawed five times prior to usual hyalruronidase treatment and sent in the first tranche, to help check whether freeze thawing SF affected the analytes detected.

**Clinical data**

Radiographic severity was defined by ordinal Kellgren and Lawrence (KL) grading for the worst affected compartment (0-4), as provided by the cohort. X-rays were not transferred or re-scored. Where individual KL grades were not available, dichotomous variables were generated, based on each cohort’s eligibility criteria, i.e. stating presence or absence of radiographic knee OA (KL≥2) and/or presence or absence of advanced radiographic knee OA (KL scores KL≥3) (S2 Table).

The Western Ontario and McMaster Universities Osteoarthritis Index (WOMAC)^[1]^, the Knee Injury and Osteoarthritis Outcome Score (KOOS)^[2]^ and various knee pain numerical rating scales were most commonly collected by cohorts. No single patient reported outcome measure was held by all participating cohorts. In a systematic review and meta-analysis carried out by consortium investigators specifically to support the harmonisation of its knee pain data^[3]^, the ability to define a harmonised knee pain threshold by analysis of Patient Acceptable Symptom State (PASS) for these subscales in these populations was explored^[3]^. These findings supported the generation of a dichotomous harmonised pain category for the purposes of the consortium work (those with unacceptable or acceptable levels of knee pain, based on a single threshold). Pain scale selection from available cohort data followed an algorithm which prioritised WOMAC pain subscale for OA cases and KOOS pain subscale for knee injury cases, followed by knee pain NRS, followed by painDETECT NRS^[4]^ where others were unavailable. These same harmonizable knee pain outcomes were also used continuously where available.

**Supplementary References**

1. Roos, E.M., M. Klassbo, and L.S. Lohmander, *WOMAC osteoarthritis index. Reliability, validity, and responsiveness in patients with arthroscopically assessed osteoarthritis. Western Ontario and MacMaster Universities.* Scand J Rheumatol, 1999. **28**(4): p. 210-5.

2. Roos, E.M., et al., *Knee Injury and Osteoarthritis Outcome Score (KOOS)--development of a self-administered outcome measure.* J Orthop Sports Phys Ther, 1998. **28**(2): p. 88-96.

3. Georgopoulos, V., et al., *The patient acceptable symptom state for knee pain - a systematic literature review and meta-analysis.* Osteoarthritis and Cartilage, 2021. **29**: p. S52.

4. Freynhagen, R., et al., *painDETECT: a new screening questionnaire to identify neuropathic components in patients with back pain.* Curr Med Res Opin, 2006. **22**(10): p. 1911-20.
