# Supplementary material for: Development of methodology to support molecular endotype discovery from synovial fluid of individuals with knee osteoarthritis: The STEpUP OA consortium
Source: PLoS One. 2024 Nov 18;19(11):e0309677. doi: 10.1371/journal.pone.0309677 (PMC11573211; doi:10.1371/journal.pone.0309677)
Supplement: Attachment — Submitted filename: Review.docx [file pone.0309677.s018.docx]

**Review**

**Methods for molecular endotype discovery in STEpUP OA**

Data availability- the data from the main study (proteomics) is not open access data. The mention of a fee to access needs more detail.

**Abstract**

The use of abbreviations should be removed in the abstract for easier reading. Indeed, some abbreviations (synovial fluid) are not even defined.

**Introduction**

Minor

Check abbreviations are defined the first time used (eg FDA).

Mixture of UK and US English, be consistent and use UK English.

**Methods**

More details on the SF preparation from collection to storage are required. What was the time from collection to centrifugation, were samples subaliquot, where samples collected into low protein bind tubes? How long were samples stored at -80 for prior to analysis? Was the supernatant removed following centrifugation? Were samples kept in a fridge at all times? Was a cold centrifuged used? Were samples with blood contamination excluded? Were samples were collected following joint lavage what buffer was used and what were the protocols?

Details of why certain criteria were used for filtering should be given from line 238-251.

Results

I cannot assess the remaining manuscript as the quality of the images is too of too poor resolution.
