# Supplementary material for: Development of methodology to support molecular endotype discovery from synovial fluid of individuals with knee osteoarthritis: The STEpUP OA consortium
Source: PLoS One. 2024 Nov 18;19(11):e0309677. doi: 10.1371/journal.pone.0309677 (PMC11573211; doi:10.1371/journal.pone.0309677)
Supplement: Attachment — Submitted filename: PLOS ONE_response to reviewers_19thJan24_final_7thFeb.docx [file pone.0309677.s019.docx]

**PLOS ONE Decision: Revision required [PONE-D-23-35180] - [EMID:a292826ab746f870]**

**Response to reviewer comments**

We thank the reviewers and editorial team for their helpful comments, questions and suggestions and detail below a point-by-point response to these. Page numbers have been given with reference to the resubmitted tracked changes manuscript and supplementary materials.

**Response to Editorial points:**

When submitting your revision, we need you to address these additional requirements.

1. **Please ensure that your manuscript meets PLOS ONE's style requirements, including those for file naming. The PLOS ONE style templates can be found at
   https://journals.plos.org/plosone/s/file?id=wjVg/PLOSOne_formatting_sample_main_body.pdf and 
   https://journals.plos.org/plosone/s/file?id=ba62/PLOSOne_formatting_sample_title_authors_affiliations.pdf**

Author Response: Thank you for highlighting these formatting requirements.

Author Action: These have now been corrected throughout the text and we hope the manuscript structure now aligns with the journal’s requirements. For example, level 1 headings are now font size 18, level 2 headings are font size 16, all headings are written in sentence case, and the correct symbols to identify sets of equal contributors have been used.

2. **We note that the grant information you provided in the ‘Funding Information’ and ‘Financial Disclosure’ sections do not match. When you resubmit, please ensure that you provide the correct grant numbers for the awards you received for your study in the ‘Funding Information’ section.**

Author Response: We apologise for this mismatch.

Author Action: We have now ensured that all grant information and numbers provided in ‘Funding Information’ match those included in the manuscript.

3. **Thank you for stating the following financial disclosure:
   "The study was supported by Kennedy Trust for Rheumatology Research (grant number: 171806), Versus Arthritis (grant number: 22473), Centre for OA Pathogenesis Versus Arthritis (grant numbers: 21621, 20205), Galapagos, Biosplice, Novartis, Fidia, UCB, Pfizer (non consortium member) and Somalogic (in kind contributions). "**

Please state what role the funders took in the study.  If the funders had no role, please state: "The funders had no role in study design, data collection and analysis, decision to publish, or preparation of the manuscript." If this statement is not correct you must amend it as needed. 
Please include this amended Role of Funder statement in your cover letter; we will change the online submission form on your behalf.

Author Response: Thank you for highlighting this omission. The funders had no role in any of these activities.

Author Action: We have added the suggested text to the cover letter and also added this statement at lines 637-643.

4. **Thank you for stating the following in the Competing Interests section:
   "YD, TAP, PH, SL, AS, NKA, DF, BM, AMV, SK, VB, JMA and VK declare no conflicts of interest. FW has received consultancy fees from Pfizer, and has a leadership role at the Medical Research Council (panel member) and Osteoarthritis and Cartilage (Associate Editor). LSL has received consultancy fees from Arthro Therapeutics AB, and is an advisory board member of AstraZeneca (non consortium member).  LJD has received consultancy fees from Nightingale Health PLC. TLV has no conflicts to declare with the exception of grant income for STEpUP OA from industry partners (see above). RAM is a shareholder of AstraZeneca. SB and JM are employees and shareholders of Novartis (consortium members). MK has received support for attending the Gordon Research Conference, OARSI meeting, International Cartilage Repair Society, Munster University, is a board member of the Dutch Arthritis Society (Chair of Visitation Board), and has a leadership role at Osteoarthritis Research Society International (Board of Directors Member). CTA has received consultancy fees from Novartis, and has received honoraria for educational purposes also from Novartis. DAW has received consultancy fees from GlaxoSmithKline plc, AKL Research & Development Limited, Pfizer Ltd, Eli Lilly and Company, Contura International, and AbbVie Inc, has received honoraria for educational purposes from Pfizer Ltd and AbbVie Inc, is a board member of UKRI (Director) and Versus Arthritis Advanced Pain Discovery Platform. "**

**Please confirm that this does not alter your adherence to all PLOS ONE policies on sharing data and materials, by including the following statement: "This does not alter our adherence to PLOS ONE policies on sharing data and materials.” (as detailed online in our guide for authors http://journals.plos.org/plosone/s/competing-interests).  If there are restrictions on sharing of data and/or materials, please state these. Please note that we cannot proceed with consideration of your article until this information has been declared.**

**Please include your updated** **Competing Interests statement in your cover letter; we will change the online submission form on your behalf.**

Author Response: Thank you for highlighting this requirement. The competing interests declared do not in themselves affect data sharing. Data sharing is covered by a consortium agreement which all members signed. Its requirements are outlined in our response to point 5.

Author Action: We have added the following statement to the Competing Interest Statement section as requested (lines 661-662): “***This does not alter our adherence to PLOS ONE policies on sharing data and materials (see also Data Availability statement)***”. However, we have also further clarified in this section the restrictions on sharing of data and/or materials as our obligations to the consortium to the response to point 5 below. We have added the updated competing interests section to our cover letter.

5. **In your Data Availability statement, you have not specified where the minimal data set underlying the results described in your manuscript can be found. PLOS defines a study's minimal data set as the underlying data used to reach the conclusions drawn in the manuscript and any additional data required to replicate the reported study findings in their entirety. All PLOS journals require that the minimal data set be made fully available. For more information about our data policy, please see http://journals.plos.org/plosone/s/data-availability.**

**Upon re-submitting your revised manuscript, please upload your study’s minimal underlying data set as either Supporting Information files or to a stable, public repository and include the relevant URLs, DOIs, or accession numbers within your revised cover letter. For a list of acceptable repositories, please see http://journals.plos.org/plosone/s/data-availability#loc-recommended-repositories. Any potentially identifying patient information must be fully anonymized.**

**Important: If there are ethical or legal restrictions to sharing your data publicly, please explain these restrictions in detail. Please see our guidelines for more information on what we consider unacceptable restrictions to publicly sharing data: http://journals.plos.org/plosone/s/data-availability#loc-unacceptable-data-access-restrictions. Note that it is not acceptable for the authors to be the sole named individuals responsible for ensuring data access.**

**We will update your Data Availability statement to reflect the information you provide in your cover letter.**

Author response: As part of legal restrictions (outlined in a consortium agreement, signed ahead of this work by all consortium members), and in line with the ethical approvals/ requirements of the contributing cohorts, the full STEpUP OA dataset cannot be made publicly available, either as files or in a public repository at any time. There are routes to access these primary datasets (discovery, replication) for bone fide research by researchers outside of the consortium after the publication of our primary analysis results. The minimal datasets upon which this data relies and all R code have been made publicly available, which allow the checking of our analysis and results whilst being in line with our consortium obligations, which we hope satisfies the journal and reviewers.

Author action: Our Data Availability Statement has been revised as follows (lines 718-724): “***The minimal datasets upon which this data relies and all R code, including the html vignette, are available at https://github.com/dengyun-git/STEpUp_QC_Paper. The full STEpUP OA dataset may be made available by application to the Data Access and Publication Group of STEpUP OA (stepupoa@kennedy.ox.ac.uk) once the primary analysis manuscript is published, in accordance with what is stipulated in our Consortium Agreement. Neither the minimal dataset nor the full STEpUP OA dataset include patient identifiable data. This may attract a fee for access to cover administrative processing***”.

6. **We note that you have included the phrase “data not shown” in your manuscript. Unfortunately, this does not meet our data sharing requirements. PLOS does not permit references to inaccessible data. We require that authors provide all relevant data within the paper, Supporting Information files, or in an acceptable, public repository. Please add a citation to support this phrase or upload the data that corresponds with these findings to a stable repository (such as Figshare or Dryad) and provide and URLs, DOIs, or accession numbers that may be used to access these data. Or, if the data are not a core part of the research being presented in your study, we ask that you remove the phrase that refers to these data.**

Author Response & action: Thank you for clarifying this. Both instances of “data not shown” have been removed (lines 351 & 356).

7. **Please include captions for your Supporting Information files at the end of your manuscript, and update any in-text citations to match accordingly. Please see our Supporting Information guidelines for more information: http://journals.plos.org/plosone/s/supporting-information.**

Author Response & action: This has been corrected (lines 947-1022).

8. **We notice that your supplementary [Figure S1-S9] are included in the manuscript file. Please remove them and upload them with the file type 'Supporting Information'. Please ensure that each Supporting Information file has a legend listed in the manuscript after the references list.**

Author Response & action: This has now been corrected.

*Comments from PLOS Editorial Office:*

1) *We note that one of the reviewers expressed concerns about the quality of the images. Although the figures appear to be of poor quality when viewed as part of the pdf, the individual figure files are of a good quality. The original figures can be accessed by clicking on the link at the top of each page with a figure to download the file. Therefore, you do not need to produce new versions of the figures at this time, although please see note 8 above.*

Author response: Thank you for clarifying this.

2) *Although there are comments from 2 reviewers below, reviewer 2 felt unable to fully assess your submission. Please ignore the comments provided by reviewer 2.*

Author response: As instructed, we have only responded to comments provided by reviewer 1.

Reviewer#1:
**1. * Data availability- the data from the main study (proteomics) is not open access data. The mention of a fee to access needs more detail.**

Author response: We apologise that our submitted manuscript did not have the minimal dataset and all R code, including the html vignette through the link provided.

Author action: The data access statement has been amended as follows: “***The minimal datasets upon which this data relies and all R code, including the html vignette, are available at https://github.com/dengyun-git/STEpUp_QC_Paper. The full STEpUP OA dataset may be made available by application to the Data Access and Publication Group of STEpUP OA (stepupoa@kennedy.ox.ac.uk) once the primary analysis manuscript is published, in accordance with what is stipulated in our Consortium Agreement. Neither the minimal dataset nor the full STEpUP OA dataset include patient identifiable data. This may attract a fee for access to cover administrative processing***”.

We can confirm that the Github link was activated ahead of submission of this revision.

**2. * Abstract: The use of abbreviations should be removed in the abstract for easier reading. Indeed, some abbreviations (synovial fluid) are not even defined.**

Author Response & action: Thank you. All abbreviations have been spelled out in full in the abstract. The abstract has also been edited to bring it down to within the allowed word count.

**3. * Introduction: Minor: Check abbreviations are defined the first time used (eg FDA).**

Author Response: Thank you. These have been checked and corrected (e.g. FDA).

**4. Mixture of UK and US English, be consistent and use UK English.**

Author Response: We note the need for consistency.

Author Action: We believe we have identified and changed all instances, e.g. standardisation, normalisation, recognise etc to UK English. For instance, ‘recognised’ (line 4), ‘localised’ (line 5), ‘utilised’ (line 28) etc.

Methods
**5. More details on the SF preparation from collection to storage are required. What was the time from collection to centrifugation, were samples subaliquot, where samples collected into low protein bind tubes? How long were samples stored at -80 for prior to analysis? Was the supernatant removed following centrifugation? Were samples kept in a fridge at all times? Was a cold centrifuged used? Were samples with blood contamination excluded? Were samples were collected following joint lavage what buffer was used and what were the protocols?**

Author response: We apologise that in trying to reduce word count, insufficient detail was provided on our laboratory methods. In addition, some of this information was available in Supplementary Methods which we appreciate may have been overlooked. It should be noted that there would be slight methodological variations across the various contributing cohorts (as SF samples were already collected). We provide here the typical approach to SF preparation, including for the host institution cohorts and also for de novo collections as per our SOP for the sample processing.

Answers to the reviewer’s specific questions are given below.

- What was the time from collection to centrifugation, were samples subaliquot, where samples collected into low protein bind tubes?
- Typical time from collection to centrifugation was 2 hours. Samples were subaliquoted into 500 ul aliquots in 2 ml cryovials (not low bind).
- How long were samples stored at -80 for prior to analysis?
- Time of storage (referred to as ‘sample age’) varied within and between cohorts and was recorded and used as a covariate in the Principal Components (PC) analysis.
- Was the supernatant removed following centrifugation?
- Yes, for centrifuged samples only the supernatants were removed and stored down. As noted, some cohorts in replication had samples that were not centrifuged and these data were handled differently.
- Were samples kept in a fridge at all times?
- No, the preference was to process as quickly as possible. In our experience, refrigerating SF makes it viscous and harder to centrifuge.
- Was a cold centrifuged used?
- A temperature controlled centrifuge was used, typically set to 20 deg C, for reason above.
- Were samples with blood contamination excluded?
- No they were not, but as listed (in tables S2 and S7), blood was graded and considered in our QC pipeline and as a covariate in the PC analysis.
- Were samples collected following joint lavage what buffer was used and what were the protocols? - Samples were never collected by joint lavage. All were collected directly from the joint, typically by needle arthrocentesis.

Author action: This additional information and other relevant points are now provided in Supplementary methods (p1-3).

**6. Details of why certain criteria were used for filtering should be given from line 238-251.**

Author response: Filters were agreed in principle in advance by our Data Analysis Group, based on typical approaches to proteomic data and are outlined in the initial QA plan (https://www.kennedy.ox.ac.uk/oacentre/stepup-oa/stepup-oa) and subsequently informed by the QC assessment of the dataset informing the data analysis plan.

Author action: We have clarified at these lines 255-257: “***For additional details, see the data analysis plan (listed in Appendix 1):*** [***https://www.kennedy.ox.ac.uk/oacentre/stepup-oa/stepup-oa***](https://www.kennedy.ox.ac.uk/oacentre/stepup-oa/stepup-oa)”.

Results
**7. I cannot assess the remaining manuscript as the quality of the images is too of too poor resolution.**
Note editorial guidance on this.

PLOS ONE: Edits requested on your submission PONE-D-23-35180R1 - [EMID:8b5bf427a9474af1]

1. **We note your current Data Availability statement is:**

**"No - some restrictions will apply;**

**The minimal datasets upon which this data relies and all R code, including the html vignette, are available at https://github.com/dengyun-git/STEpUp_QC_Paper. The full STEpUP OA dataset may be made available by application to the Data Access and Publication Group of STEpUP OA once the primary analysis manuscript is published, in accordance with what is stipulated in our Consortium Agreement. Neither the minimal dataset nor the full STEpUP OA dataset include patient identifiable data. This may attract a fee for access to cover administrative processing."**

**However, the URL you provided, https://github.com/dengyun-git/STEpUp_QC_Paper, did not turn up any results.**

**Before we can proceed, please confirm that that the provided URL is active and publicly accessible.**

**Additionally, please provide non-author contact details, such as an email address, for the Data Access and Publication Group of STEpUP OA to which data access requests can be sent.**

**We will update your Data Availability statement on your behalf using the information you provide.**

Author response: We apologise for the error. Access to the GitHub repository was checked and confirmed prior to re-submission but since then, the account was made private in error. The repository is now publicly available and can be accessed at the url previously provided (<https://github.com/dengyun-git/STEpUp_QC_Paper>).

At the editor’s request, we have provided an email address to which data access requests can be submitted (lines: 719-722: “***The full STEpUP OA dataset may be made available by application to the Data Access and Publication Group of STEpUP OA (stepupoa@kennedy.ox.ac.uk) once the primary analysis manuscript is published, in accordance with what is stipulated in our Consortium Agreement***”). For consistency, we have amended this in the text and provided the updated text in the cover letter.

2. **We notice that your manuscript file was uploaded on Oct 31 2023. Please can you upload the latest version of your revised manuscript as the main article file, ensuring that does not contain any tracked changes or highlighting.  This will be used in the production process if your manuscript is accepted. Please follow this link for more information:** [**http://blogs.PLOS.org/everyone/2011/05/10/how-to-submit-your-revised-manuscript/**](http://blogs.plos.org/everyone/2011/05/10/how-to-submit-your-revised-manuscript/)

Author response & action: Both a clean version and tracked changed version of the revised manuscript have now been uploaded.

3. **Your ethics statement should only appear in the Methods section of your manuscript. If your ethics statement is written in any section besides the Methods, please delete it from any other section.**

Author response & action: We have removed the Ethics Statement at the end of the manuscript (lines 731-733).
