# Supplementary material for: Development of methodology to support molecular endotype discovery from synovial fluid of individuals with knee osteoarthritis: The STEpUP OA consortium
Source: PLoS One. 2024 Nov 18;19(11):e0309677. doi: 10.1371/journal.pone.0309677 (PMC11573211; doi:10.1371/journal.pone.0309677)
Supplement: Attachment — Submitted filename: PLOS ONE Revision_06062024_final.docx [file pone.0309677.s020.docx]

**PLOS ONE Decision**: Revision required [PONE-D-23-35180R1] - [EMID:8d32a3fbdc941e1f]

**Manuscript Number**: PONE-D-23-35180R1

**Response to reviewer comments**

We thank the reviewers and editorial team for their helpful comments, questions and suggestions and detail below a point-by-point response to these. Page numbers have been given with reference to the resubmitted tracked changed manuscript and supplementary materials.

**Reviewer 3:**

**It’s nice to see that state-of-the-art technology is being used to analyze OA-SF involving a really large number of patients to unravel the disease pathology.
Following are my comments:**

1. **Comment 1**

**It is an interesting thought that OA is actually not a single disease, but involves different types such as immune-mediated inflammation, mechanically-mediated inflammation (‘mechanoflammation’), low/failed tissue repair and cellular senescence may exist as distinguishable endotypes. However, all of these endotypes have significant proportion of overlapping pathophysiology which tend to blur the distinction between them. How the authors plan to address this complex issue?**

Author response: Our aim in STEpUP OA was to establish whether there are distinct molecular endotypes discernible from synovial fluid (SF) proteins. Our methodology, which is shown in full in the next paper (now in preprint: <https://www.medrxiv.org/content/10.1101/2024.06.05.24308485v1>) (and can be reviewed in our analysis plan shown here: <https://www.kennedy.ox.ac.uk/oacentre/stepup-oa>) is to use unsupervised k-means clustering on the SomaScan data. However, to the authors comment, an inherent limitation of this type of analysis is that the analysis is biased towards finding endotypes if they have distinct rather than overlapping proteome profiles. Continuous protein profiles, or ones that are strongly overlapping, may still be clinically meaningful and this can be tested. We have mentioned in the discussion that we will use k means clustering of the data in the primary analysis of STEpUP OA. We feel that discussing the limitations of this is best left to the next manuscript as we are not discussing it further here.

Author Action: We have added additional text to help clarify (***lines 558-560****:* “*Our next step, the primary analysis of this dataset, seeks to answer definitively whether there are distinct discernible molecular endotypes,* ***by unsupervised k means clustering****, in this common, yet poorly understood disease”*).

2. **Comment 2**

**There is a lot of dynamics of protein variation in as per the grades of the OA (usually measured on KL grade scale) is this study is going to take the grades in account?**

Author Response: The primary focus of this manuscript was the development and validation of a methodology for large-scale analysis of synovial fluid proteins in knee OA which was inclusive of multiple radiographic grades. The grading approach employed for all cases was the KL grading system where it was available (in 766 OA cases). For the others we were able to categorise them into non-advanced (KL 0-2) and advanced radiographic OA (KL 3-4) by cohort eligibility criteria. A wide range of KL grades were intentionally included, to be as generalisable as possible. The association of differing radiographic grades with protein abundance is already included as part of our primary analysis plan (<https://www.kennedy.ox.ac.uk/oacentre/stepup-oa>); as above, the link to this plan is included at various points in this manuscript. A description of the cohorts and the variables included in the analysis, including their range in radiographic severity by KL grade can be found in S1 Table (final column).

Author Action: We have added additional information on the radiographic scoring systems used in the methods with reference to S1 Table (***lines 156-158****:* “***Characteristics of cohorts and participant samples, including details of the radiographic scoring employed (Kellgren-Lawrence^[58]^ grading where available), are described in S1 Table with core variable definitions also provided*** *(S2 Table & Supplementary methods)****.***”).

We have added the following reference, cited as [58], to the text:

Kellgren, J.H. and J.S. Lawrence, *Radiological assessment of osteo-arthrosis.* Ann Rheum Dis, 1957. **16**(4): p. 494-502.

3. **Comment 3**

**If purpose of this communication is only to report a method/protocol for largescale analysis of synovial fluid proteins and not to report any of the endotypes, the paper title is misleading. Please find an appropriate title.**

Author Response: Thank you. We do wish to make it clear that this is primarily a methodology paper and not the primary outcome of the analysis.

Author Action: We have changed the title to: “*Development of* ***methodology to support*** *molecular endotype discovery from synovial fluid of individuals with knee osteoarthritis: the STEpUP OA Consortium*”

4. **Comment 4**

**If the authors are using ssDNA aptamers/SOMAmers, for harvesting proteins form synovial fluid. They must assure that the clinically obtained SF to be free from DNA and RNA. There is lot of DNA in synovial fluid (Ref. DOI: 10.1002/art.1780240905). Similarly, there are large number of micro and long non-coding RNAs present in the SF. How is the system free from their interreference?**

Author Response: We appreciate this comment. We believe that free DNA is unlikely to lead to significant assay interference as the SOMAmer technology relies on high affinity binding with specificity and selectivity for protein targets. Low affinity binding nucleic acids are removed during the assay process. As a result, any residual nucleic acids present in the SF are unlikely to interfere with the binding of SOMAmers to their protein targets.

Author Action: A comment regarding the presence of non-specific nucleic acids in the SF has now been added to the Discussion (***lines 535-538****:* “***Free nucleic acids in biological fluids such as SF could potentially interfere with the SomaScan assay. However, this is unlikely as the aptamers used for protein identification are designed to be high affinity for that protein with low affinity binders being competed off through application of an unlabeled polyanion competitor ^[72]^.”).***

Reference [72] has been added to the text:

Williams, S.A., et al., *Plasma protein patterns as comprehensive indicators of health.* Nat Med, 2019. **25**(12): p. 1851-1857.

5. **Comment 5**

**How the protocol reduce/deplete hugely abundant proteins like albumin, globulin form SF?**

Author response:

There are no steps for depletion of highly abundant proteins. One of the advantages of SomaLogic technology is that aptamer binding quantification can be carried in complex samples across a wide range of protein concentrations. This is achieved by dividing the sample into three dilutional ‘bins’ (1:5, 1:200 and 1:20,000) which enables high abundant proteins to be examined separately from low abundant ones.

Author Action: We have added to Methods the following (***lines 126-127****:* “*Samples were assayed* ***in three ‘dilution bins’ (to allow accurate quantification of high and low abundant proteins simultaneously)*** *on the SomaLogic SomaScan Discovery Plex V4.1 by SomaLogic, in Boulder, US.)*”).

6. **Comment 6**

**SF contain several proteases including very active tryptase and chymase, elastase etc. How have you prevented their action thereby loss of proteins on storage?**

Author response: We are unable to control fully for such activities within the SF. To minimise these, we recommended strict adherence to standardised protocols for sample handling and storage to reduce protease activation and protein degradation. This included minimising freeze-thaw cycles (aliquoting individual samples prior to freezing), and ensuring consistent storage conditions to prevent fluctuations in temperature that may affect protease activity. Processing in the laboratory was also standardised to reduce enzymic activity at the time of analysis. Furthermore, we looked carefully for confounding technical factors that could have reflected poor protein stability (or active degradation) by including “age of sample”, “number of freeze-thaws”, “spun status” in our quality analysis planning. We report these findings in this manuscript for this reason and incorporated filters for proteins that were poorly stable on freeze thaw/storage as likely to be less informative.

*Author Action: We have added the following text to the Methods: (****lines 542-545****: “…without a unified pre-specified sample processing protocol. This made distinguishing technical and biological variation,* ***including through protease activity****, difficult (**as was the case for the intracellular protein score, which could reflect either variation in joint biology or variation in sampling handling).”*

7. **Comment 7**

**Why the Picture quality is so low?**

Author response & action: High quality images were submitted but likely compressed during first review phase. We will ensure that higher quality images are available in the final version.
