# Supplementary material for: Development of methodology to support molecular endotype discovery from synovial fluid of individuals with knee osteoarthritis: The STEpUP OA consortium
Source: PLoS One. 2024 Nov 18;19(11):e0309677. doi: 10.1371/journal.pone.0309677 (PMC11573211; doi:10.1371/journal.pone.0309677)
Supplement: Attachment — Submitted filename: PLOS ONE Revision_19072024_final.docx [file pone.0309677.s021.docx]

**PLOS ONE Decision**: PLOS ONE Decision: Revision required [PONE-D-23-35180R2]

**Full Title**: Development of methodology to support molecular endotype discovery from synovial fluid of individuals with knee osteoarthritis: the STEpUP OA Consortium

**Response to reviewer comments**

We thank the reviewers and editorial team for their helpful comments, questions and suggestions and detail below a point-by-point response to these. Page numbers have been given with reference to the resubmitted tracked changed manuscript and supplementary materials.

**Editor**:
**Reviewers responded that the revise generally addresses the issues. However, reviewer 4 pointed out two remaining issues.** **And Editor has a confirmation question.**

1) **Comment 1**

**On page P12, lines 193-195, authors refer to Cip as the logarithmic value of the protein concentration of sample i. Since the addition of the logarithm of a number is the logarithm of the multiplication of that number, isn't IPSi the logarithm after the addition?

That is, IPSi=logΣdpCip**

Author response:

We thank the editor for pointing this out. The equation is correct, but the text immediately before and after it missed out the word "log" in two places, which made it inaccurate. We have fixed this now.

Author action:

We have made the following two changes to the text:

- line 191: "weighted sum of ***log*** protein concentrations"
- line 194: "difference in ***log*** concentration"

**Reviewer 3**:

**The authors have satisfactorily addressed all the questions raised, expect following two points:**

2) **Comment 1**

**Interreference of DNA form SF: the SF DNA can bind to the single stranded aptamers/somamers and therefore the related proteins may be underrepresented. Similarly, the micro and lnRNAs may also bind to the ssDNA aptamers and therefore may interfere the protein detection. The SF may be treated with DNAse and RNAse before proceeding.**

Author response:

We thank the reviewer for the comment. By incorporating DNase and RNase treatments, it may have been possible to reduce potential interference from DNA and RNA. However, our adopted protocol was in accordance with standard procedures using SomaScan (King *et al*. 2020; Rydén *et al*. 2024). It is worth noting that the binding affinity of aptamers is enhanced by chemical modification of the DNA.

- Rydén M, *et al*. Exploring the Early Molecular Pathogenesis of Osteoarthritis Using Differential Network Analysis of Human Synovial Fluid. Mol Cell Proteomics. 2024 Jun;23(6):100785. doi: 10.1016/j.mcpro.2024.100785. Epub 2024 May 14. PMID: 38750696.
- King JD *et al*. Joint Fluid Proteome after Anterior Cruciate Ligament Rupture Reflects an Acute Posttraumatic Inflammatory and Chondrodegenerative State. Cartilage. 2020 Jul;11(3):329-337. doi: 10.1177/1947603518790009. Epub 2018 Jul 22. PMID: 30033738; PMCID: PMC7298591.

Author action:

We have added the following text to the discussion (see lines: 535 to 540): “Free nucleic acids in biological fluids such as SF could potentially interfere with the SomaScan assay. However, this is unlikely as the aptamers used for protein identification, ***which are chemically modified DNA molecules***, ***are high affinity for that protein allowing*** low affinity binders ***to be*** competed off through application of an unlabeled polyanion competitor^[72]^. ***It might have been possible to reduce interference further by pre-treating SF with DNAse or RNAse, but this is not considered standard for SomaScan analysis^[73, 74]^***.”.

We have also added the above two references to the text (line 540).

3) **Comment 2**

**SF protease may hydrolyse proteins upon long-term storage. The protease action has nothing to do with any freeze-thaw cycles or temperature fluctuations. I may suggest that the authors may use commercially available cocktail of proteinase inhibitors containing TPCK, TLCK, PMSF etc. This will effectively reduce protein degradation in long-term storage.**

Author response: We agree that this needs to be included as a comment for considering design of future studies although in this case all of our samples were pre-collected so this was not an option at the time of study development.

Author action:

We have added the following line to the discussion (see lines: 547 to 549): “***Intrinsic protease activity within the fluids may have contributed to confounding associated with sample age. Addition of protease inhibitors at the time of collection could be something to consider in future sample collections, but is generally not the norm***.”

**Reviewer 4**:

The authors appropriately responded to all of the comments the reviewer had raised. This manuscript now can be publishable.
